# A Review on the Role of Vitamin D in Asthma

**DOI:** 10.7759/cureus.1288

**Published:** 2017-05-29

**Authors:** Niloufer S Ali, Kashmira Nanji

**Affiliations:** 1 Family Medicine, The Aga Khan University

**Keywords:** vitamin d, asthma, asthma morbidity, vitamin d deficiency, vitamin d insufficiency, immunomodulation

## Abstract

Asthma, a major public health issue, is one of the most common diseases affecting millions of population globally. It is a chronic respiratory disease characterized by increased airway inflammation and hyper-responsiveness. Vitamin D is of particular interest in asthma due to its immunomodulatory effects. Serum 25-hydroxyvitamin D is found to be associated with a wide range of pulmonary diseases, including viral and bacterial respiratory infections, asthma, and cancer. Several researches have reported positive associations between vitamin D and asthma. On the other hand, others have reported contrasting effects of vitamin D on asthma.

This review provides an examination of current epidemiologic and experimental evidence of a causal association between vitamin D status and asthma or asthma exacerbations, including its probable protective mechanism. Most of the evidence regarding vitamin D and asthma is reported by observational studies. Therefore, results from the experimental trials of vitamin D supplementation are important as they can provide evidence for future recommendations about the significance of vitamin D for asthma. Moreover, the trials can be effective in assessing the correct dosage and safety of vitamin D supplementation when given in diverse age groups such as children, teenagers, and adults for prevention and treatment of asthma.

## Introduction and background

Asthma is one of the most common chronic diseases and its prevalence has increased worldwide in the last few decades affecting approximately 300 million people. This poses an immense burden on healthcare resources [1].

Asthma is a prolonged inflammatory disorder which is related with hyper responsiveness of the airways and leads to symptoms such as wheezing, dyspnea, chest tightness, and cough mainly at night or early in the morning [2]. The factors particularly responsible for asthma are not very clear because of its different presentation in both adults and children [3]. Interleukins (IL)-4, IL-5, and IL-13 (T-helper cell type-2 cytokines) are regulated in the asthmatic airway and are related with increased eosinophilia [3], mast cell degranulation [4], and increased levels of immunoglobulin E (IgE) [3-4]. The complex interaction between cells and inflammatory mediators and impairment of immunogenic tolerance promotes airway injury. This process is known as airway “remodeling” [5]. This process of remodeling involves hypertrophy of smooth muscle, hyperplasia of epithelial goblet cell, and deposition of airway extracellular matrix proteins which may lead to increase airflow obstruction and finally causing the respiratory symptoms [6].

Several dietary hypotheses have been proposed in context with asthma [7-8] and among them vitamin D status is of particular interest. Studies suggest that there is a probable relationship between vitamin D status and asthma-related symptoms presumably via the immune-modulatory effects of vitamin D [5, 8].

### Risk factors of asthma

Numerous risk factors of asthma can be divided into host and environmental factors.

Host Factors

Genetic: Several genes can be related to the pathogenesis of asthma. Four major areas have been focused in this regard; production of IgE antibodies, airway hyper-responsiveness, and inflammatory mediators (cytokines, chemokines), growth factors and the ratio between Th1 and Th2 immune responses [5-8].

Obesity: Obesity has been associated with the development of asthma. However, the biologic causality has not been yet proven [7, 9].

Gender: Prevalence rates of asthma are different among males and females [8-9]. Childhood asthma is found to be common in boys than girls and by adulthood, this is reversed [10]. Reasons for this gender differences are not clear. However, the lung size is smaller in males than in females at birth but larger in adulthood [8-9].

Environmental Factors

Allergens: The role of allergens in the development of asthma in children is still unclear. It relies on the allergen, the dose, the time of exposure and the age of the child, and genetics can also play a role [10].

Infections: Evidence suggests that respiratory infections in early childhood may have a protective role against the development of asthma and studies have shown that they may develop asthma in later life [8, 11].

Tobacco smoke: Studies reveal that exposure to tobacco both prenatally or after birth can increase the risk of developing asthma-like symptoms in early childhood [12].

Outdoor/Indoor pollutants: The role of pollutants in the development of asthma is less well defined. It has been observed that increased levels of outdoor or indoor pollutions can aggravate asthma symptoms [12-13].

Diet: Studies have shown that infants fed with commercial cow’s milk formula etc. have a higher frequency of wheezing illnesses in early childhood as compared to breast milk feeding infants [11, 13].

### Pathophysiology

Narrowing of airway causes asthma and its symptoms [2]. Following are the factors which contribute to the development of this physiologic change [9-10].

In response to multiple broncho-constrictor, mediators and neurotransmitters the airway smooth muscle gets contracted and this leads to narrowing of the airway; this can be retreated by bronchodilators [10].

### Diagnosis of asthma

It involves a careful process of history taking, physical examination, and diagnostic testing. The family history of asthma and atopic disease can also assist in diagnosing asthma [12, 14].

Physical Examination

The common abnormal physical finding on the examination is wheezing on chest auscultation. In severe cases, a silent chest can be found because of limitation of airflow and ventilation [2]. However, the other symptoms include: difficulty in speaking, drowsiness, tachycardia, cyanosis and use of accessory muscles and intercostal recession [14]. Confirmation of asthma is based on two additional elements [2, 14].

Spirometry: It is the preferred method of diagnosis of airflow obstruction. Peak flow measurements alone should not be used to diagnose asthma due to wide variability in predicted peak expiratory reference values. It may be more useful in monitoring patient’s symptoms and response to therapy [14].

Exclusion of alternative diagnosis: Common acute causes of wheezing in children must be omitted before diagnosing asthma in children [14].

### Management

The management of asthma involves controlling the clinical symptoms and maintaining it for a long term.

Pharmacologic Treatment

It can be divided into two categories: controllers and relievers.

Controllers: They are basically a preventive measure to keep asthma under control and taken daily on long term basis. Sometimes to reach clinical control add-on therapy with another class of controller is preferred over increasing the dose of inhaled corticosteroids [15-16].

Relievers: They are fast acting beta2 agonists and are the most effective medication for quick relief of bronchospasm in adults and children [15-16].

## Review

### Vitamin D

Vitamin D is a fat-soluble nutrient which is a modulator of calcium absorption and bone health. It also plays an important role in immune regulation and in respiratory infections [10, 17]. Serum 25OHD is the top indicator of overall vitamin D status since it reﬂects the intake of vitamin D from dietary sources intake as well as sun exposure; it also accounts for the adaptation of vitamin D from adipose stores in the liver [17]. Though, there are no guidelines on optimum levels of serum 25OHD. However, vitamin D deﬁciency is deﬁned as 25OHD level of less than 50 nmol/L (20 ng per millilitre) [18]. When 25OH D levels are between 50 and 75 nmol/L (20–30 ng per millilitre) it is an indicator of vitamin D insufﬁciency [18]. Moreover, 25OHD levels of 75 nmol/L (30 ng per millilitre) to 100 nmol/L suggest normal vitamin D levels. Vitamin D intoxication is defined as, serum levels of 25(OH) D are greater than 150 ng/ml [18].

### Vitamin D epidemiology

Vitamin D deficiency has a high incidence worldwide. It is estimated that almost half of the healthy people population are 25-hydroxy-vitamin D (25-OH D) deficient [19]. Insufficient sun exposure or pigmented skin and inadequate dietary intake are the main causes of these low levels of 25-OH D [19]. A study conducted in the US on 9,757 subjects found that approximately 9% of participants had vitamin D deficiency and 61% had vitamin D insufficiency [20]. The common factors of vitamin D deficiency are increasing age, being female, Mexican-American ethnicity, obesity, and reduced dairy intake [19-20].

### Vitamin D deficiency in South Asia

Sunlight exposure helps vitamin D production and in South Asia, almost all the days are sunny [21-22]. However, still, there is a high prevalence of vitamin D deficiency in these countries due to other underlying factors such as lack of proper diet, inadequate calcium intake, social culture and customs that require the elderly, children and females to be confined in the house, limiting their exposure to sunlight [20-21]. It has been reported that the prevalence of this deficiency ranges from 69% to 82% in Indian population [23-24]. This is not limited to the Asians living in India and Pakistan [5, 9], but even the immigrants (UK, Denmark, and Norway) have this deficiency [23-24].

### Vitamin D and immune system

Vitamin D plays a significant part in inborn and adaptive immunity; however, it is not completely understood [9, 25]. It is reported that over 900 genes are regulated by vitamin D [26]. This action of vitamin D has gained immense acceptance after the discovery of vitamin D receptor (VDR). Several studies have shown that vitamin D prevents the increase of CD41 T-cells [25-26] and decreases the creation of Th1 cytokines IL-17 [10, 25-27]. However, due to alterations in target cells timing and quantity of vitamin administration, studies have shown contrasting results. Evidence suggests that innate immunity is activated by the production of anti-microbial peptide LL-37 by macrophages [25-27]. The adaptive immune system increases the production of T-cells and modifies the functions of antigen-presenting cells (APCs), of dendritic cells. In addition, vitamin D has been revealed to improve and inhibit IL-4 production by naive T-cells [25-27].

### Vitamin D and asthma

It has been suggested that modernization and westernization have led to vitamin D deficiency among world population. Since, the majority of the population spends time indoors away from sun exposure, leading to vitamin D deficiency [21, 27].

The role of vitamin D in asthma is not yet clear. Few cross-sectional surveys had suggested a probable link between asthma and vitamin D [27-28]. Studies have concluded that decreased level of serum 25(OH)D is correlated with an increased prevalence, hospitalization, and increased emergency visits along with declined lung function and increased airway hyper-responsiveness in asthmatic children [27, 29]. Clinical trials conducted in recent times have shown the protective influence of vitamin D supplementation among asthmatic patients [30-32]. In addition, increased intake of vitamin D during pregnancy has an influence on asthma in children and adults [33-34].

Evidence from the researches concludes that asthma exacerbations and resistance to common therapies are some of the major challenges to reduce asthma-related morbidity and mortality [29-34]. Studies are suggestive in support of a role of vitamin D in both these aspects [29-34].

Evidence from Observational Studies

A cross-sectional survey on 75 Italian asthmatic children found that the prevalence of vitamin D-deficiency was 53.3% [28]. In another survey from North America, 17% of asthmatics had vitamin D deficiency and a positive correlation was observed between vitamin D levels and lung function [29]. A study conducted by Thuesen, et al. on 4,999 Danish adults reported contrasting results and concluded that 25(OH)D levels do not have any effect on the development of asthma and allergic symptoms [35]. A cohort study revealed that low serum 25(OH)D levels at the age of 6 years predicted asthma-associated symptoms (uncontrolled asthma and lung function) at 14 years of age [36].

The data from observational studies has shown a parallel relation between vitamin D and asthma outcomes [36-39]. Although, it is challenging to formulate a relationship between them due to certain limitations of these studies such as the presence of bias (selection bias), confounders (physical activity, sex, age, etc.) and small sample size. All of the above-mentioned limitations might have caused a spurious relation between vitamin D and asthma.

Cohort studies suggest that lower maternal dietary intake of vitamin D can result in the development of asthma morbidity and wheeze in children [40-42]. However, a major limitation of these studies was that they assessed vitamin D intake using food frequency questionnaires rather than directly measuring serum concentrations.

A study conducted by Gale, et al. shared that children whose mothers had serum 25(OH)D concentrations above 75 nmol/L had five times more risk of developing asthma at 9 years [33]. Studies conducted in Finland and Japan on more than 750 mother-child pair have found that dietary vitamin D intake during pregnancy is inversely related to the incidence of wheeze among children [41-42]. Another study concludes that serum 25(OH)D during late pregnancy was not related with maternal vitamin D status and risk of childhood asthma [43]. A study conducted by Zosky, et al. found that maternal vitamin D deficiency was positively related with asthma at 6 years in males (OR: 3.03; 95% CI: 1.02-9.02) [40].

Comparison between the results of the studies should be done with caution, as these studies had a number of limitations, for instance, short follow-up duration, significant rates of attrition, and lack of validated measures for measuring serum vitamin D.

A study done in New Zealand revealed that low blood 25(OH) D levels were not associated with asthma incidence [44].

The relation between higher 25(OH)D levels and reduction in the incidence of asthma is contradictory and studies depicting a direct role for vitamin D in the development of asthma are required.

### Vitamin D and asthma pathogenesis

A study conducted by Gupta, et al. was the pioneer research in concluding that serum vitamin D levels were found to be lowest among children with steroid resistant asthma (STRA) [45] with reduced lung function, increased corticosteroid use, and asthma exacerbations. The probable reason for this is because low vitamin D levels increase the airway smooth muscle (ASM) and reduce lung function in severe asthma [45].

Evidence from Observational Studies

Vitamin D is only used for primary prevention for asthma but it also has a role in asthma exacerbation. A cross-sectional study of 616 asthmatic children in Costa Rica between the ages of 6 and 14 years, reported that vitamin D deficiency/insufficiency was prevalent in 28% of the children and increased levels of vitamin D were related with reduction in asthma exacerbations and reduced visits to emergency department (OR: 0.05; 95% CI: 0.004-0.71) [20]. A 4-year longitudinal study was conducted by Brehm, et al. to establish causal relations between serum vitamin D levels and asthma exacerbations in 1024 North American asthmatic children with mild to moderate asthma [25]. This study confirmed that less levels of vitamin D (<30 ng/mL) had increased risk of asthma exacerbations (OR: 1.5; 95% CI: 1.1-1.9) [25]. In addition, the study also concluded that children with vitamin D insufficiency had higher chances of asthma exacerbation [25].

Another cross-sectional study conducted on 560 children ages between 6 and 14 years with asthma in Puerto Rico reported that children with vitamin D insufficiency were 2.6 times more at risk of developing asthma exacerbations (OR: 2.6; 95% CI: 1.5-4.9) [37]. A recent study conducted on 70 adult asthmatic patients and 20 healthy controls by Shahin, et al. [46], concluded that serum vitamin D level was significantly decreased in asthmatic patients (19.88 ± 9.6 ng/ml) as compared to the control group (33.5 ± 6.1 ng/ml) [46] (Table 1).

**Table 1:** Observational studies of vitamin D and asthma morbidity

Author	Year	Study Design	Study Sample	Outcome Measures	Findings
Brehm [20]	2009	616 children diagnosed with asthma in Costa Rica	Mild to persistent asthma	Asthma exacerbations	Increased levels of vitamin D was related with decreased hospitalizations, use of steroids and airway hyperresponsiveness
Chinellato [28]	2011	Cross-sectional survey of 75 children	Controlled and uncontrolled asthma	Spirometry, asthma controls	Vitamin D levels were positively correlated with asthma control
Gale [33]	2008	Birth cohort study of 596 mother-child pairs	Intake of vitamin D during pregnancy	Serum 25(OH)D	Maternal vitamin D levels were linked with higher risk of asthma
Hollams [34]	2011	Cohort of 2,834 pairs (mothers & child)	Subjects with and without asthma	Physician diagnosed asthma	Vitamin D status at 6 years of age was predictive of asthma symptoms at 14 years
Thuesen [35]	2016	Longitudinal study of 4,999 Danish population aged 30 to 60 years; 3,032 had completed follow-up at 5 years	Subjects with and without asthma	Physician diagnosed asthma	Serum vitamin D levels do not have influence on asthma among adults
Freishtat [36]	2010	Case-control study of 106 African American subjects 6 to 20 years of age	Subjects with and without asthma	Physician diagnosed asthma	Vitamin D deﬁciency was related to asthma
Brehm [37]	2012	560 Puerto Rican kids	Subjects with and without asthma	Physician diagnosed asthma	Vitamin D insufficiency was related to increased odds of asthma exacerbation (OR: 2.6; 95% CI: 1.5–4.9)
Miyake [39]	2010	Birth cohort of 1,002 Japanese mothers and child	Maternal consumption of vitamin D	Food-frequency questionnaire	Increased intake of vitamin D was accompanied with a lower risk of wheeze
Zosky [40]	2014	Prospective birth cohort study with children		Serum 25(OH)D on 16-20 weeks of gestation	Maternal vitamin D deficiency had impact on asthma in males
Devereux [29]	2014	Case-control study of 160 adults in the United Kingdom	Subjects with and without asthma	Physician diagnosed asthma	No association between vitamin D level and asthma
Erkkola [42]	2009	Birth cohort in Finland with 3,565 (mother-children) with 1,669 children	Susceptibility to Type I diabetes	Maternal food-frequency questionnaire	Increased maternal vitamin D intake lowers risk of asthma
Morales [43]	2012	Prospective cohort of 1724 children in Spain	Maternal intake of vitamin D	Maternal plasma 25(OH)D	Increased maternal intake of vitamin D was associated with a lower risk of lower respiratory infections
Camargo [44]	2011	Cohort of 922 newborns in New Zealand, children		Cord blood 25(OH)D	Higher 25(OH)D resulted in reduced risk of respiratory infections and wheezing but had no effect on incidence of asthma
Shahin [46]	2017	Two groups. First group included 70 adult asthmatic patients and 20 healthy adult control group	Subjects with and without asthma	Vitamin D status in patients with asthma and its relation to disease control and severity	Vitamin D levels were significantly decreased in asthmatic patients (19.88 ± 9.6 ng/ml) as compared with the healthy control group (33.5 ± 6.1 ng/ml)

Evidence from Clinical Trials

Clinical trials have provided suggestions that vitamin D has a protective role against asthma exacerbations. A randomized, double-blind 6-month clinical trial conducted by Majak, et al. found that children receiving supplemental 500 IU/d vitamin D had lower risk of asthma exacerbations when triggered through acute respiratory infections [31]. Another randomized, double-blind, parallel arm clinical trial by Urashima, et al. on 217 school children found that there was a lower risk of asthma exacerbations after vitamin D3 supplementation [30]. Other trial on 100 asthmatic children revealed that monthly doses of 60,000 IU of vitamin D can significantly decrease the number of exacerbations (p = 0.011) and it also resulted in the reduction of steroids (p = 0.013) and emergency visits (p = 0.015) [32]. A study conducted in 2016 in which Japanese schoolchildren with asthma (n = 89) were randomly assigned to either receive vitamin D (n = 54) or placebo (n = 35). At 2 months, asthma control was significantly more improved in the vitamin D group as compared to the placebo group (p = 0.015) [47].

Some trials on vitamin D did not show statistical significance and remained inconclusive. A recent trial by Jensen, et al. found that preschool-aged children with asthma received 100,000 IU vitamin D3 or placebo, followed by 400 IU vitamin D3 daily for 6 months revealed that there was no significant difference in the Serum 25OHD between the groups [48]. Another study conducted to determine whether vitamin D supplementation reduces cold symptom occurrence and severity among asthmatics found that despite achieving 25(OH)D levels of 41.9 ng/ml, vitamin D supplementation had no effect on the rate of colds between groups [49]. A study conducted by Martineau, et al. found that intermittent bolus dose of vitamin D3 to a daily low-dose regimen was associated with increased risk and duration of upper respiratory infections [50] (Table 2).

**Table 2:** Trials of vitamin D and asthma morbidity GINA: Global Initiative for Asthma; RCT: Randomized clinical trial.

Author	Year	Study Design	Study Sample	Outcome Measures	Findings
Urashima [30]	2010	RCT of 217 school children	All school children with subgroup with clinically diagnosed asthma	Asthma exacerbations	Vitamin supplements reduced the risk of severe asthmatic symptoms
Majak [31]	2011	Randomized, double-blind, parallel arm clinical trial 6-month experimental study of vitamin D3 (500 IU/d) as adjuvant therapy to IC among 48 Polish children	Newly diagnosed asthma	Asthma exacerbations	Children receiving vitamin D supplementation had lower incidence of asthma exacerbations as compared to other groups
Yadav [32]	2014	Randomized double blinded placebo controlled trial on 100 children 1 group received vitamin D3, 60,000 IU/month for 6 months versus placebo	Asthmatic children	Asthma exacerbations and number of emergency visits	Vitamin D has a role in bronchial asthma
Tachimoto [47]	2016	Treatment (n = 54): vitamin D3 800 IU/day orally for 2 months. Control (n = 39): daily oral placebo for 2 months	Asthmatic children (6 to 15 years)	Changes in asthma control levels defined by GINA. Changes in asthma control levels judged by the childhood ACT	Asthma control was significantly improved in vitamin D group versus placebo group (p = 0.015). Childhood asthma control test (CACT) scores were also significantly improved in vitamin D group
Jensen [48]	2016	Active intervention (n = 11): 100,000 IU vitamin D3 oral bolus at baseline, followed by 400 IU vitamin D3 IU orally daily. Control intervention (n = 11): oral placebo at baseline, followed by 400 vitamin D3 IU orally daily	Asthmatic preschool children (1 to 5 years)	Mean group change in total serum 25(OH)D from baseline to 3 months	Group difference in serum 25OHD (7.2 nmol/L; 95% CI: (13.7-28.1) was not significant; 100% vs 54.5% (intervention versus control) had serum 25OHD ≥75 nmol/L
Denlinger [49]	2015	Cholecalciferol (100,000 IU load plus 4,000 IU/d) for 28 weeks or placebo	Adults with asthma	Cold symptom severity, which was assessed using daily scores on the 21-item Wisconsin Upper Respiratory Symptom Survey	Vitamin D supplementation had no effect on the average peak scores (62.0 [95% CI: 55.1–68.9; placebo] and 58.7 [95% CI: 52.4–65.0; vitamin D]; p = 0.39)
Martineau [50]	2015	Six 2-monthly oral doses of 3 mg vitamin D3 (n = 125) or placebo (n = 125) over 1 year	Adults with asthma	Asthma exacerbations and URI	No significant association to first severe exacerbation (HR: 1.02, 95% CI: 0.69-1.53, p = 0.91) or first URI (HR: 0.87, 95% CI: 0.64-1.16, p = 0.34)

These data certainly are suggestive of a relation of vitamin D in asthma and can be useful as an adjunct therapy to overcome steroid resistance in severe asthma. There are many studies on the influence of vitamin D on responses to infection, but there are none or limited studies to determine the causal role of vitamin D deficiency during acute asthma exacerbation.

### Future perspectives

Most of the observational researches have demonstrated the benefit of vitamin D supplementation in asthmatic patients. Nevertheless, to address the limitation of temporality in the observational studies, clinical trials are required to establish the association between vitamin D and asthma.

Trials with larger sample sizes are needed to provide the evidence of causality for vitamin D and asthma. These trials will also be helpful in establishing the appropriate route, dose, and safety of vitamin D supplementation for prevention and treatment of asthma.

It is also imperative to realize the protective mechanism of vitamin D against asthma. The suppression of Th2 immune response explains the protective effect of vitamin D alongside asthma. Nonetheless, there is a need to identify evidence between vitamin D and atopic reactions.

There is no evidence to suggest that asthmatic patients should be screened for vitamin D deficiency or insufficiency. However, the high-risk group (obese patients and elderly of African-American ethnicity who have limited sun exposure) must be screened for this deficiency.

Further researches are needed on the molecular level of vitamin D receptor to explain the role of dietary vitamin D in the prevention and management of asthma.

## Conclusions

Vitamin D can be effective as an adjunct therapy in the management of asthma. The higher levels of vitamin D, in fact, are needed to prevent various health problems. Regardless of the growing evidence about the association of vitamin D with asthma and asthma exacerbation, the results of the studies are not reliable. Several studies have supported the view that vitamin D deficiency is the cause of the global asthma epidemic. On the other hand, another set of studies has proposed that vitamin D supplements are responsible for the asthma epidemic.

However, larger trials with huge sample sizes are required to define the role of vitamin D in asthma and answer questions that whether vitamin D supplementation will be more effective in the high-risk vitamin D deficient group or not.
